# DMM Outstanding Paper Prize 2019 winner: Alessandro Bailetti

**DOI:** 10.1242/dmm.046672

**Published:** 2020-08-14

**Authors:** Rachel Hackett

**Affiliations:** The Company of Biologists, Bidder Building, Station Road, Histon, Cambridge CB24 9LF, UK

## Abstract

Disease Models & Mechanisms (DMM) is delighted to announce (with apologies for the delay) that the winner of the DMM Prize 2019 is Alessandro Bailetti, for his paper entitled ‘Enhancer of Polycomb and the Tip60 complex repress hematological tumor initiation by negatively regulating JAK/STAT pathway activity’ ( Bailetti et al., 2019). The prize of $1000 is awarded to the first author of the paper that is judged by the journal's editors to be the most outstanding contribution to the journal that year. To be considered for the prize, the first author must be a student or a postdoc of no more than 5 years standing.

## Outstanding contribution

Dr Alessandro Bailetti grew up in Pisco, a small port city in the south of Peru. In his family's farm, he saw his father and grandfather use basic genetics to improve the crops and cattle of their farm. His family immigrated to northwest Florida in the United States in 2004. After finishing high school, Alessandro decided to attend his local community college, Pensacola State College (PSC; Pensacola, FL, USA). Quickly, Alessandro became involved in campus life and joined the Phi Theta Kappa Honors Society. Alessandro graduated from PSC in 2009 with an associate's degree in mathematics. He was awarded the very competitive Jack Kent Cooke Undergraduate Transfer Scholarship and transferred to Cornell University (Ithaca, NY, USA). He joined the Lin laboratory to study Jagged1 and Notch signalling in mammalian neurogenesis. He was awarded the National Science Foundation (NSF)-Cornell University Undergraduate Research Fellowship to undergo his undergraduate research. His honours research focused on the roles of Jagged1 in neurogenesis during mouse development. In collaboration with a PhD student, Chris Blackwood, part of his work was published in Frontiers of Biology (Blackwood et al., 2020).

**Figure:**
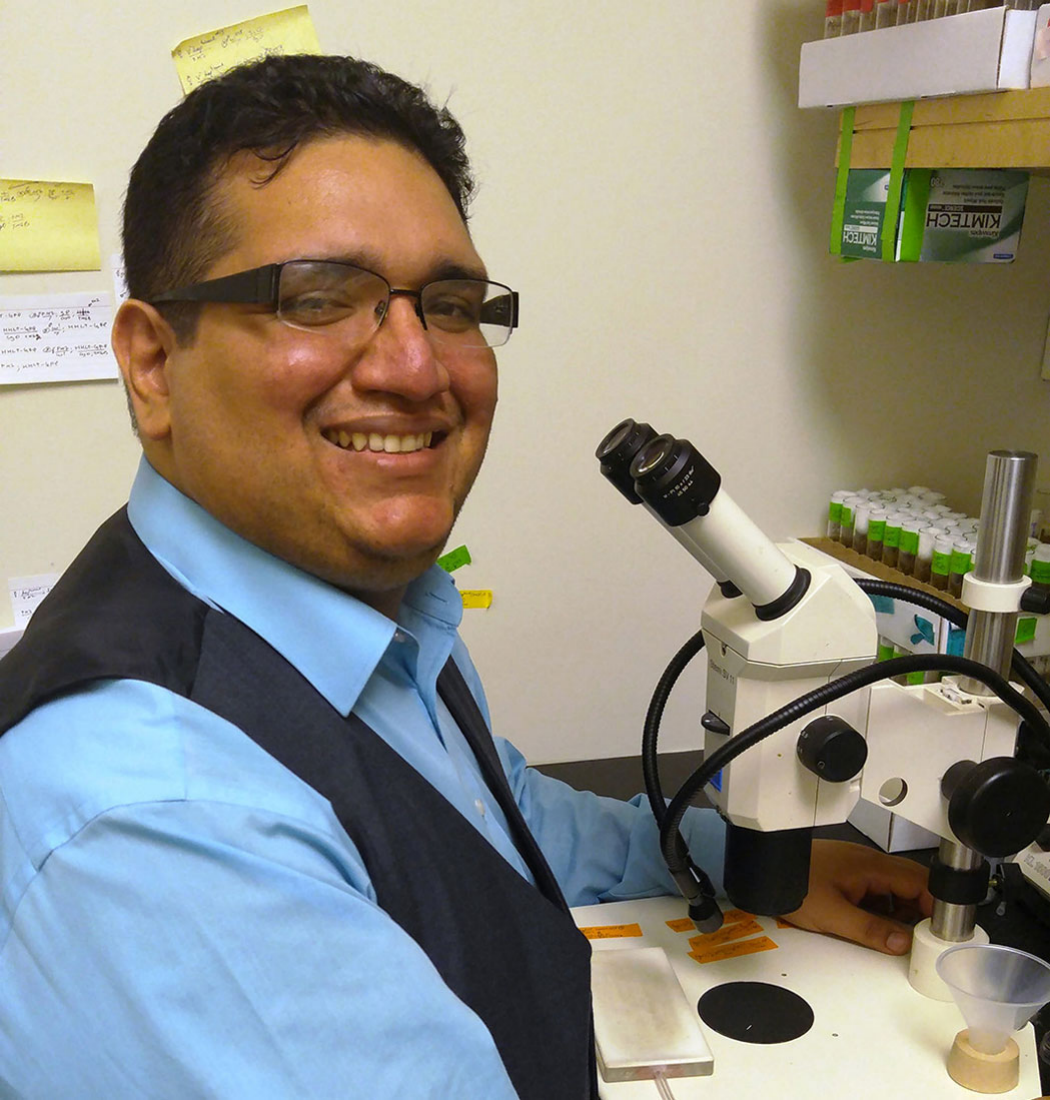
**Alessandro Bailetti**

**Box 1. DMM Prize 2019 shortlist**

**Winner:**

Enhancer of Polycomb and the Tip60 complex repress hematological tumor initiation by negatively regulating JAK/STAT pathway activity.

Alessandro A. Bailetti, Lenny J. Negrón-Piñeiro, Vishal Dhruva, Sneh Harsh, Sean Lu, Aisha Bosula and Erika A. Bach.

Disease Models & Mechanisms (2019) 12, dmm038679. doi:10.1242/dmm.038679

Also shortlisted by our Editor team:

Adenosine and hyaluronan promote lung fibrosis and pulmonary hypertension in combined pulmonary fibrosis and emphysema.

Scott D. Collum, Jose G. Molina, Ankit Hanmandlu, Weizhen Bi, Mesias Pedroza, Tinne C. J. Mertens, Nancy Wareing, Wang Wei, Cory Wilson, Wenchao Sun, Jayakumar Rajadas, Paul L. Bollyky, Kemly M. Philip, Dewei Ren, Rajarajan A. Thandavarayan, Brian A. Bruckner, Yang Xia, Michael R. Blackburn and Harry Karmouty-Quintana.

Disease Models & Mechanisms (2019) 12, dmm038711. doi:10.1242/dmm.038711

ApoE-associated modulation of neuroprotection from Aβ-mediated neurodegeneration in transgenic Caenorhabditis elegans.

Edward F. Griffin, Samuel E. Scopel, Cayman A. Stephen, Adam C. Holzhauer, Madeline A. Vaji, Ryan A. Tuckey, Laura A. Berkowitz, Kim A. Caldwell and Guy A. Caldwell.

Disease Models & Mechanisms (2019) 12, dmm037218. doi:10.1242/dmm.037218

RDH10 function is necessary for spontaneous fetal mouth movement that facilitates palate shelf elevation. Regina M. Friedl, Swetha Raja, Melissa A. Metzler, Niti D. Patel, Kenneth R. Brittian, Steven P. Jones and Lisa L. Sandell.

Disease Models & Mechanisms (2019) 12, dmm039073. doi:10.1242/dmm.039073

Early detection of pre-malignant lesions in a KRASG12D-driven mouse lung cancer model by monitoring circulating free DNA.

Callum P. Rakhit, Ricky M. Trigg, John Le Quesne, Michael Kelly, Jacqueline A. Shaw, Catrin Pritchard and L. Miguel Martins.

Disease Models & Mechanisms (2019) 12, dmm036863. doi:10.1242/dmm.036863

Leptin induces muscle wasting in a zebrafish kras-driven hepatocellular carcinoma (HCC) model.

Qiqi Yang, Chuan Yan, Xu Wang and Zhiyuan Gong.

Disease Models & Mechanisms (2019) 12, dmm038240. doi:10.1242/dmm.038240

Modelling pancreatic β-cell inflammation in zebrafish identifies the natural product wedelolactone for human islet protection.

Luis Fernando Delgadillo-Silva, Anastasia Tsakmaki, Nadeem Akhtar, Zara J. Franklin, Judith Konantz, Gavin A. Bewick and Nikolay Ninov.

Disease Models & Mechanisms (2019) 12, dmm036004. doi:10.1242/dmm.036004

He graduated with honours with a bachelor's degree in biological sciences from Cornell University in 2012. He was awarded the NSF Graduate Research Fellowship and the Jack Kent Cooke Graduate Scholarship to pursue his PhD degree at New York University (New York, NY, USA). Alessandro joined Dr Erika Bach's laboratory for his doctoral studies. In the Bach laboratory, Alessandro worked on using *Drosophila* as a model for myeloproliferative neoplasms (MPNs). MPN patients carry a JAK2 mutation that increases JAK/STAT signalling and proliferation of myeloid lineage cells. A mutation in the fly JAK gene (*hop*) called *hop^Tum^* increases JAK/STAT activity and myeloid-like cells, a similar phenotype to that found in MPN patients (Anderson et al., 2017).

In collaboration with a postdoc, Dr Abigail Anderson, Alessandro performed a deficiency screen looking for modifiers in the tumour burden in the JAK hyperactive mutant *hop^Tum^*. They and colleagues found 32 enhancers and 11 suppressors of the tumor burden (Anderson et al., 2017). Alongside Dr Anderson, Alessandro validated one of the enhancers, *expanded*, which is part of the Hippo pathway. They and colleagues found a novel role of the Hippo pathway in the circulating haemocytes, concluding that Hippo activity contributes to the suppression of haemocyte proliferation, and that Yki activation, owing to Hippo mutations or ectopic expression of *yki*, increases haemocyte proliferation (Anderson et al., 2017).

Dr Bailetti also explored a second enhancer from the deficiency screen, Enhancer of Polycomb [E(Pc)] (Anderson et al., 2017; Bailetti et al., 2019). Unlike its name suggests, E(Pc) is a member of the Tip60 complex. The Tip60 complex transfers acetyl groups to lysine residues, which is reversed by the action of lysine deacetylases (KDACs). Bailetti and colleagues found that depletion of E(Pc) in the haematopoietic system increased the *hop^Tum^* tumour burden (Bailetti et al., 2019). More interestingly, depletion of E(Pc), Tip60 and other members of the Tip60 complex in a healthy wild-type animal was sufficient to lead to development of lamellocytes and tumour formation. This showed that the Tip60 complex was necessary for tumour suppression and lamellocyte differentiation in flies. In addition, Alessandro showed that depletion of E(Pc) and Tip60 in the haematopoietic system increased the JAK/STAT activity without increasing the expression of its ligands, JAK or STAT. Finally, he showed that depletion of E(Pc) or the inhibition of Tip60, using KDAC inhibitors, increased the Hop protein level. This elucidated that the role of E(Pc) through the Tip60 complex in the haematopoietic system was to attenuate Hop protein levels and, hence, JAK/STAT activity (Bailetti et al., 2019).

Alessandro is an avid promoter of equality and inclusion in science. He has been a member of multiple mentoring programmes aimed at increasing minority students in the science, technology, engineering and mathematics (STEM) field. Alessandro was a mentor and director of the Clear Directions mentoring program at New York University designed to help high-school minority students discover careers in the STEM field. He is also an active member of the Research Experience for Peruvian Undergrads (REPU) programme that helps current undergraduate students in Peru find international research experience. Dr Bailetti is currently a postdoctoral fellow at Stanford University (Stanford, CA, USA) in Dr Anthony Oro's laboratory. His current research aims to understand how the genomic landscape changes and contributes during stem cell differentiation to skin.
